# Brainless Walking: Animal Gaits Emerge From an Actuator Characteristic

**DOI:** 10.3389/frobt.2021.629679

**Published:** 2021-04-29

**Authors:** Yoichi Masuda, Keisuke Naniwa, Masato Ishikawa, Koichi Osuka

**Affiliations:** ^1^ Department of Mechanical Engineering, Osaka University, Suita, Japan; ^2^ Research Institute for Electronic Science, Hokkaido University, Sapporo, Japan

**Keywords:** legged robot, quadruped robot, motion control, gait analysis, motors, autonomous decentralized control, oscillator, vibration

## Abstract

In this study, we discovered a phenomenon in which a quadruped robot without any sensors or microprocessor can autonomously generate the various gait patterns of animals using actuator characteristics and select the gaits according to the speed. The robot has one DC motor on each limb and a slider-crank mechanism connected to the motor shaft. Since each motor is directly connected to a power supply, the robot only moves its foot on an elliptical trajectory under a constant voltage. Although this robot does not have any computational equipment such as sensors or microprocessors, when we applied a voltage to the motor, each limb begins to adjust its gait autonomously and finally converged to a steady gait pattern. Furthermore, by raising the input voltage from the power supply, the gait changed from a pace to a half-bound, according to the speed, and also we observed various gait patterns, such as a bound or a rotary gallop. We investigated the convergence property of the gaits for several initial states and input voltages and have described detailed experimental results of each gait observed.

## 1 Introduction

Most of the legged animals have the ability to adaptively select their gait patterns according to their speed (Alexander, 1984). Although the mechanism of the gait selection in animals is still unclear, conventional animal experiments have provided us with some knowledge. A study investigating the oxygen consumption of running horses (Hoyt and Taylor, 1981) showed that horses choose an efficient gait depending on their running speed. The result suggests that animals choose an energetically appropriate gait to survive in nature with limited resources. A study comparing the characteristics of the transition points in various animals’ gait (Heglund et al., 1974) showed that the ratio of stride frequency to body weight at the transition points from trot to gallop was linear in logarithmic coordinate. The result indicates that the control mechanism for selecting motion patterns depends on the basic dynamics of the body rather than on animal species. As described above, the control principle of selecting the gait patterns of animals is not only energy efficient but also common to animals with different morphologies.

If we can imitate the ability of gait generation and selection in animals, the locomotor ability of legged robots will be improved. However, since animal gait patterns emerge as a result of complex interactions between the brain, body, and environment, it is difficult to determine which factors dominate gait generation and selection. In order to understand the principles of animal locomotion, researchers have conducted a variety of animal experiments and proposed gait generation models from various perspectives ranging from neural circuits to body dynamics. The neural network called the central pattern generator (CPG), which is located within the animal’s spinal cord, is widely known as a mechanism for generating motor patterns (Grillner, 1985). Experiments using the hind limbs of cats (Brown, 1911) observed that they could generate alternating muscle activity between flexors and extensors without sensory information from the muscles. In other experiments focusing on the generation of motor patterns, decerebrated cats generated a walking gait on a treadmill (Rossignol, 2000), and observed the gait transitions from walking to fast walking and galloping (Stuart and Hultborn, 2008; Armstrong, 1988). After the discovery of the CPG, some researchers have been tried to replicate and understand the CPG (Marder and Bucher, 2001; Ijspeert, 2008; Aoi et al., 2017). In connectionist approaches based on mathematical neuron models, multi-layer CPG models (McCrea and Rybak, 2008) have been proposed, which are capable of generating several periodic motions. More abstract CPG models using oscillators, such as the Kuramoto oscillator (Kuramoto, 1975) and Matsuoka oscillator (Matsuoka, 1985), were adopted as control laws for robots (Ijspeert, 2008; Maufroy et al., 2010; Aoi et al., 2013; Fukuoka et al., 2015).

Although the CPG has the ability to generate motor patterns by itself, several simulations and robotic experiments have shown that the spinal reflex system, which is simpler than the CPG, can also generate motor patterns by itself. It is theoretically shown that the two stretch reflex system and the physical (nonneural) interaction between the muscles stabilize the alternating motion patterns between the antagonistic muscles in a one-joint neuromechanical model (Masuda et al., 2019). In a simulation study of bipedal walking (Geyer and Herr, 2010), a human model in the sagittal plane with some reflexes, including neural connections between the left and right limbs, generated a stable gait pattern. In a simulation study of quadrupedal gait (Ekeberg and Pearson, 2005), although a three-dimensional model of the cat’s hind limbs has no neural connection between the left–right limbs, it generated a stable gait using a four-phase reflex rule. This reflex rule has also been implemented in a musculoskeletal robot (Rosendo et al., 2014). Another musculoskeletal robot with simpler reflexes (Masuda et al., 2020) has developed fast running motions by using a reciprocal excitatory reflex between the hip and knee extensors, even though there was no neural coupling between the left and right limbs, or explicit design of the walking phases and the leg trajectories. As a robot that generate multiple motion patterns, a quadruped walking robot (Owaki and Ishiguro, 2017), which uses a reflexive rule described by an oscillator model, generated various gait patterns, such as walk, trot, and gallop, depending on the speed, using only physical (nonneural) interactions between the limbs. These experiments suggest that the physical interactions between the limbs through the body and the ground play a greater role in the generation of adaptive gait.

In addition, some simpler gait generation phenomena have been reported, in which gait patterns emerge from body–environment dynamics alone, without even using reflexes. A prime example is the passive dynamic walker, which generates a bipedal gait through interaction with the ground and gravity (McGeer, 1990; Collins et al., 2005). As passive walkers that generate gait patterns, experiments with passive quadrupedal walkers (Nakatani et al., 2009) and passive bipedal walkers (Owaki et al., 2008) have shown a variety of adaptive gait generations. Furthermore, as a motor control approach that utilizes the body, which is slightly different from passive walking, there are examples of gait generation that utilize the vibration modes of the robot body. In a simple robot with a body made of a flexible U-shaped curved beam and a single DC motor (Reis et al., 2013), the continuous rotation of the DC motor generates multiple gait patterns by entraining the coupled body–environment dynamics into a resonant mode. These results are a good example of the gait selection due to the interaction between the dynamics inherent in the robot body and the environment, without the need for control by the nervous system. Thus, it has been shown that animals and robots can generate gaits not only by CPGs but also by different levels of subsystems, such as simpler reflex systems and body dynamics alone. This suggests that the animal’s ability to generate gait is not provided by a single functional module such as CPGs, but by parallel overlapping gait generation mechanisms that complement each other’s functions. Therefore, there may be additional unknown mechanisms behind the phenomenon of gait generation and selection.

**FIGURE 1:**
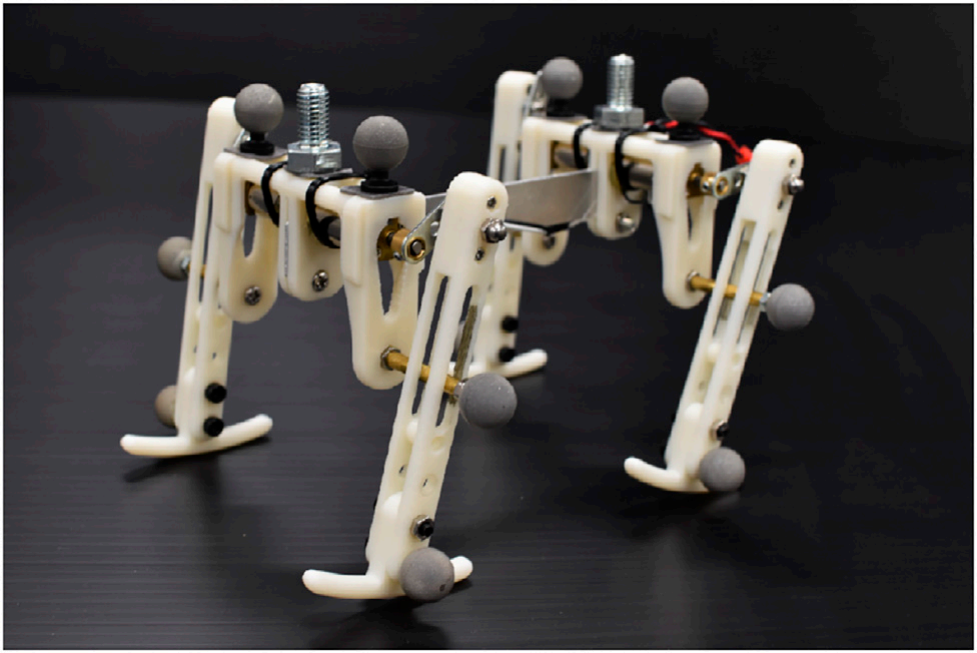
Overview of the robot.

This article describes a novel gait generation mechanism that we discovered from a different perspective than previous studies. The major contribution of this study was discovering a phenomenon in which a quadruped robot without any sensors or microprocessor can autonomously generates the various gait patterns of animals using actuator characteristics and select the gaits according to the speed. The robot, shown in Figure 1, has one DC motor on each limb and a slider-crank mechanism connected to the motor shaft. Since each motor is directly connected to a power supply, the robot only moves its foot on an elliptical trajectory under a constant voltage. In other words, this robot does not have any computational equipment, such as sensors or microprocessors. Nevertheless, when we applied a voltage to the motor, each limb begins to adjust its gait autonomously and finally converged to a steady gait pattern. Furthermore, by raising the input voltage from the power supply, the gait changed from a pace to a half-bound according to the speed, and also we observed various gait patterns, such as a bound or a rotary gallop. We investigated the convergence property of the gaits for several initial states and input voltages and describe detailed experimental results of each gait observed. A prototype of this robot was presented at an international conference on robotics (Masuda et al., 2017b). The analysis of the synchronization phenomenon of multiple DC motors in fundamental systems is described in Masuda et al., 2017a.

## 2 Quadruped Robot

This section describes a quadruped robot that can generate gait patterns and perform adaptive gait selection even though it has no sensors, microprocessor, or other computing resources.

### 2.1 Mechanical Structure

**FIGURE 2:**
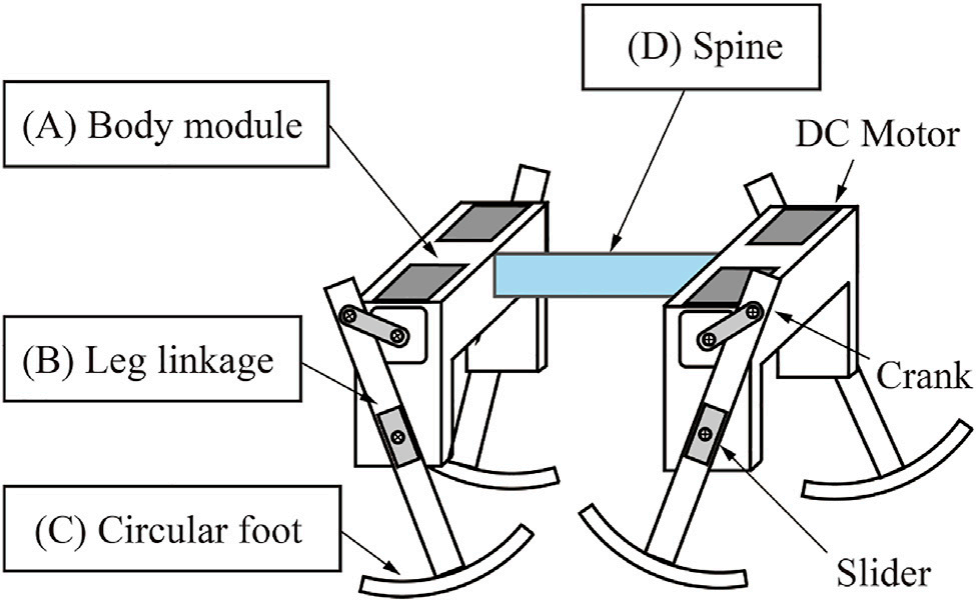
Structure of the robot.

**FIGURE 3:**
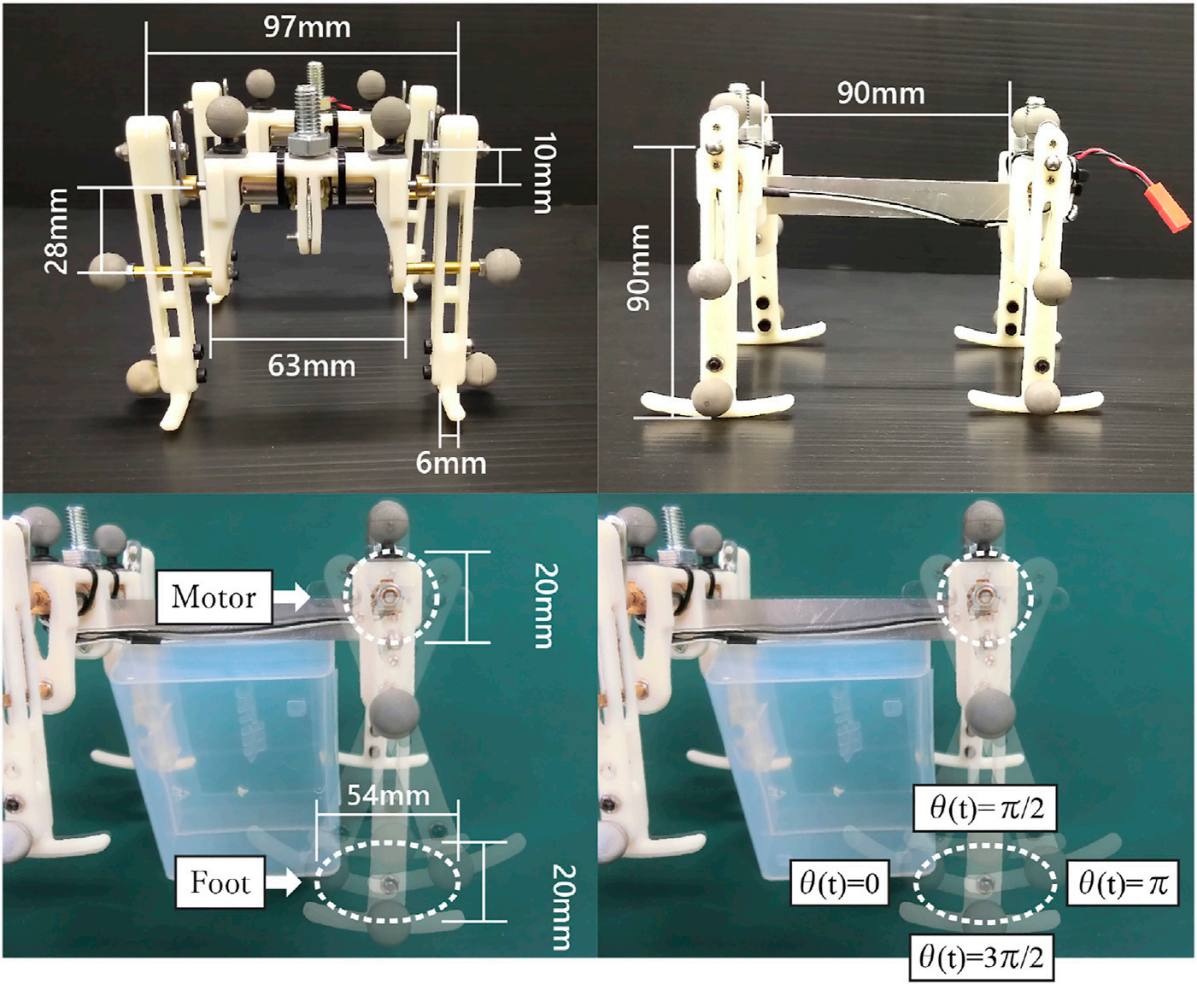
Measurements of the robot. We designed the distance between the crank tip and foot tip to be 90 mm, and the foot width is 6 mm. The right figure shows the foot trajectory of the robot. The blue box in the center of the image is a stand for fixing the robot in the air.

Figure 2 shows the structure of the quadruped robot. The robot consists of fore and hind body modules, and the modules are connected with a spine. Figure 3 shows the measurements of the robot. We designed the distance between the crank tip and foot tip to be 90 mm, and the foot width is 6 mm. The total mass of the robot, which includes the two body modules and four limbs, is 183 g.

Each module has right and left limbs, and each limb has a slider-crank mechanism connected to the shaft of a geared DC motor (Pololu 75:1 Micro Metal Gearmotor HP). Figure 2A shows the structure of the body modules. Each body module has two DC motors, so the robot has four motors in total, and all the motors are directly connected to a stabilized power source in parallel.

Each limb of the robot consists of a slider-crank mechanism. Figure 2B shows the robot limb. The limb has one degree of freedom, and the motor just rotates continuously under a constant voltage. Therefore the motor generates only an elliptical trajectory as it turns the crank, as shown in Figure 3 right. The lengths of the major and minor axes of the elliptical trajectory are 54 and 20 mm, respectively.

We embedded a circular foot for smooth touch-down and take-off of the limb. Figure 2C shows the circular foot. The shape of the foot was designed as the arc of a circle. The radius of the arc of the circular foot is 85 mm, and the foot length is 49 mm.

## 3 Controller Inherent in Motor Dynamics

When the robot walks, each DC motor moves the foot on an elliptical trajectory under a constant voltage; thus, it generates a fixed foot trajectory. In spite of such a simple configuration, this robot generates a gait according to the locomotion speed, while adjusting the phases of the motors. The key to the phase adjustment is the torque–velocity characteristics of DC motors, as described below.

### 3.1 Modeling of DC Motor

Equation of motion and circuit for a DC motor with a constant voltage *V* applied is given by

$$J\ddot{\theta }\left(t\right)+D\dot{\theta }\left(t\right)=K_{T}i\left(t\right)+\frac{\tau \left(t\right)}{n},$$ (1)

$$V=L_{M}\dot{i}\left(t\right)+Ri\left(t\right)+nK_{E}\dot{\theta }\left(t\right),$$ (2)

where *i*(*t*) is the input amplitude, *θ*(*t*) is the motor angle, τ(t) is the load torque applied to the output shaft of the gearbox, *n* is the gear ratio, *L*_*M*_ is the inductance, and *R* is the armature resistance. *J* and *D* are the inertia and the viscous resistance coefficient, respectively, including the rotor, gears, and shafts. *K*_*T*_ and *K*_*E*_ are the proportionality coefficient between torque–current and the electromotive force constant.^1^ Note that since $K_{T}=K_{E}$ from the reciprocity theorem, we write $K:=K_{T}=K_{E}$ in the following.

Assuming that the inductance *L*_*M*_ of the motor are negligible, the dynamics of the motor Eqs 1, 2 can be rewritten as follows:

$$n\varepsilon J\ddot{\theta }\left(t\right)=\left(\omega -\left(n\varepsilon D+1\right)\dot{\theta }\left(t\right)\right)+\varepsilon \tau \left(t\right),$$ (3)

where ω:=V/nK is the rotation speed at no load and $\varepsilon :=R/n^{2}K^{2}$ is the motor constant.

Finally, assuming that the inertia *J* and viscous resistance coefficient *D* are sufficiently small,^2^ the relationship between the torque–velocity characteristics of the motor, that is, the angular velocity $\dot{\theta }\left(t\right)$ and the load torque τ(t) can be written as follows:

$$\dot{\theta }\left(t\right)=\omega +\varepsilon \tau \left(t\right).$$ (4)

From the right-hand side of Eq. 4, in the absence of a disturbance torque τ(t) from the environment, the angular velocity converges to a constant $\dot{\theta }\left(t\right)=\omega$ that is proportional to the input voltage. In addition, when an external load τ(t)<0 from the environment is applied to a DC motor, the rotation speed of the motor $\dot{\theta }\left(t\right)$ increases or decreases. The interesting point of this research is that the torque–velocity characteristics, which cause inconvenience in general motor control, are utilized as a control law to adjust the motor phases in response to external forces.

### 3.2 Phase Adjustment Function Emerged From Motor and Linkage

As introduced above, thanks to the torque–velocity characteristics of the motors, the interaction between the motors, body, and the environment changes the walking motion of the robot. Next, in order to understand the general behavior of the motors in a walking robot, we model the limb linkage with a DC motor.

The structure of the load torque τ(t) changes depending on the ground contact condition. For example, when a robot’s foot is in the air, the dynamics are dominated by the inertia of the limb linkage, the rotor, and the shaft of the motor. On the other hand, when the foot is on the ground, the limb linkage is supported by the ground and the dynamics of the robot body dominates. However, the weight of the limb linkage was only 12 g compared to the body weight of 178 g. Therefore, in this study, we focus on the ground reaction force during the stance phase, which is the largest influence that the motor receives from the environment, and consider how the ground reaction force may affect the motor phase during walking.^3^

Moreover, during the stance phase, the robot receives forces from various directions depending on the condition of the environment (unevenness of the floor and friction coefficient) and the robot’s motion (gait, body posture, and relative velocity to the environment). Since these external forces emerge from the complex interaction between the body, motor, and the environment, detailed modeling of floor reaction forces is not possible and does not make sense. However, we know that the reaction force the robot receives is typically an upward force under gravity. Therefore, we discuss the general effect of a typical ground reaction force: a vertical upward force to the ground.

**FIGURE 4:**
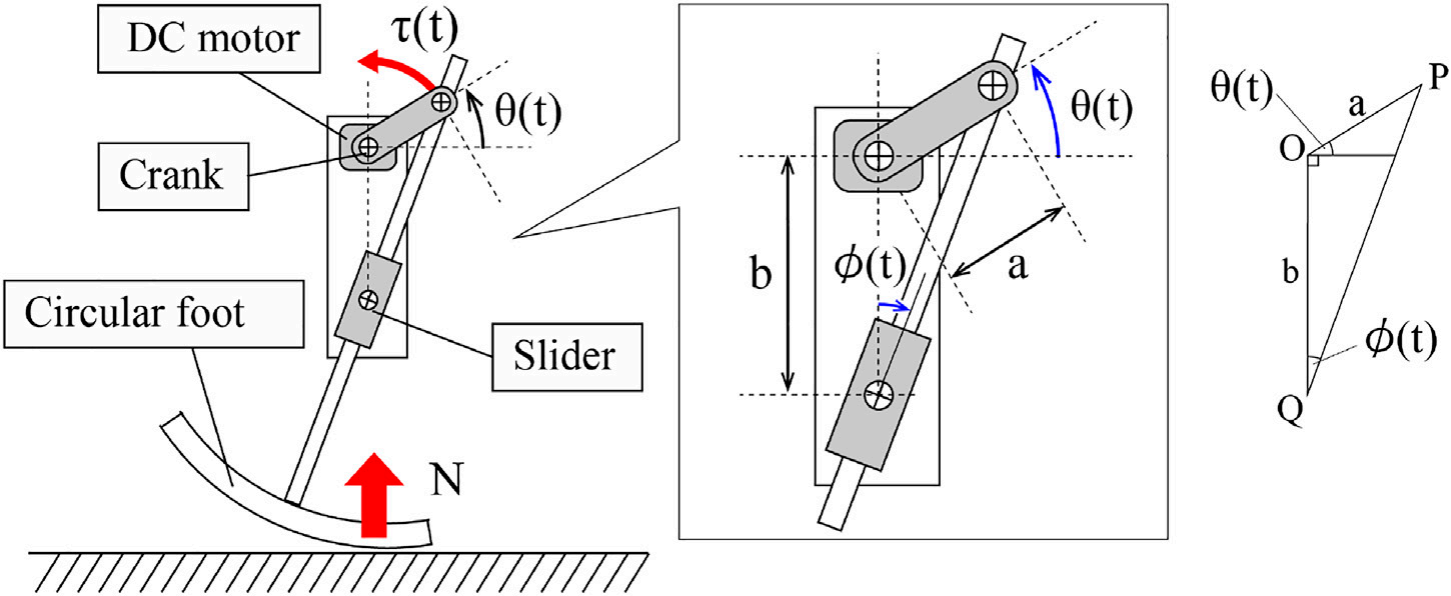
Slider-crank mechanism of the limb.

In order to discuss the general effect of the vertical ground reaction force on the rotation of the motor, we assume that the body posture of the robot is constant with respect to the ground. Moreover, we also assume that the ground contact point is nearly under the motor shaft O and the slider shaft Q, thanks to the circular foot, as shown in Figure 4. From this assumption, the axial load from the slider shaft Q to the tip of the crank *p* can be written as N(t)cosϕ(t). Here, N(t) is the vertical ground reaction force received by the foot of the robot, and ϕ(t) is the relative angle between the limbs and the body. Therefore, the torque from the tip of the crank *p* to the motor shaft is τ(t)=aN(t)cosϕcos(ϕ(t)+θ(t)). Here, since cos(ϕ(t)+θ(t))=b/asinϕ(t) by kinematics of the linkage, the load torque of the motor, τ(t), can be written as follows:

$$\tau \left(t\right)=\frac{b}{2}N\left(t\right)\text{sin}2\phi \left(t\right),$$ (5)

where *a* and *b* is the length of the crank and the distance from the motor shaft to the slider, respectively.

Then, the motor model Eq. 4 can be rewritten using Eq. 5 as follows:

$$\dot{\theta }\left(t\right)=\omega +\varepsilon \frac{b}{2}N\left(t\right)\text{sin}2\phi \left(t\right).$$ (6)

Here, note that the leg angle ϕ(t) is a function of the motor phase: ϕ(θ,t)=arctan(cosθ(t)/sinθ(t)+b/a). Here, let us see the second term on the right side of Eq. 6. Interestingly, since the state variable θ(t) is in the second term, the load torque b/2N(t)sin2ϕ(t,θ) from the environment can be interpreted as a state feedback rule of the motor angle θ(t). That is to say, DC motors Eq. 6 are the physical devices that have all the three functions of “actuate, sense, and control” necessary for adaptation to the environment.

Now, let us consider the behavior of a quadruped robot when the ground reaction forces are applied to the limbs. In Eq. 6, we consider the case where a ground reaction force N(t)>0 is applied to the foot. When the motor phase is −π/2≤θ(t)<π/2, the load torque becomes b2N(t)sin2ϕ(t,θ)>0 ^4^ and the motor speed increases, and when the motor phase is π/2≤θ(t)<3π/2, the motor speed decreases. Under a sufficiently large ground reaction force N(t)≫0, Eq. 6 has a stable equilibrium point θ=π/2 and an unstable equilibrium point θ=3π/2.^5^ Thanks to the torque–velocity characteristics of the motor and the limb, which supports the body weight and stays around the equilibrium point θ=π/2, and when the external force decreases, the motor quickly drives the limb to kick the ground.

## 4 Experimental Result

**FIGURE 5:**
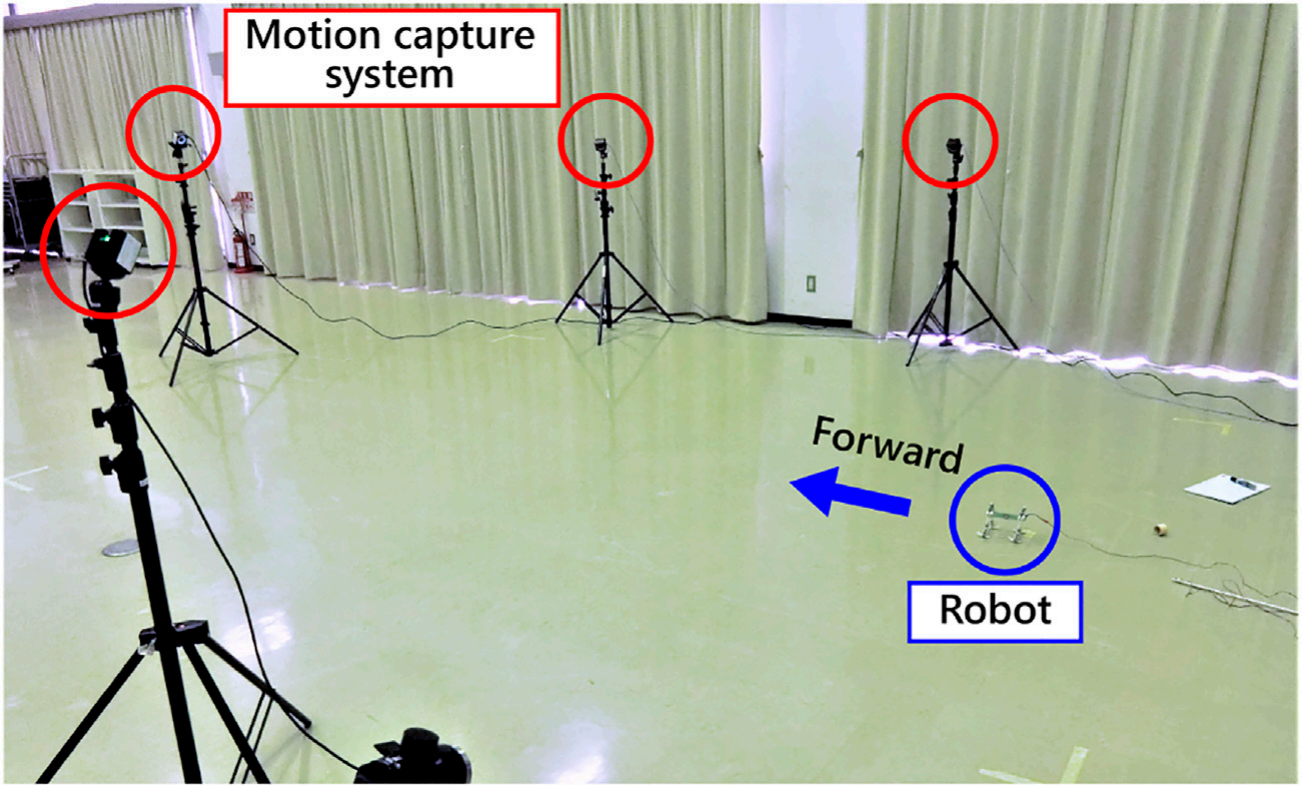
Experimental setting.

In this section, we report on the speed-adaptive gait generation and selection due to the torque–velocity characteristics of the motor. Figure 5 shows the experimental setting. All of the motors were connected to a power supply in parallel. In the following, we call the limbs [Left-Fore, Right-Fore, Left-Hind, and Right-Hind] as [LF, RF, LH, and RH]. The phases of the motors and the robot posture are calculated from data with a motion capture system (OptiTrack Prime13, NaturalPoint). Markers are set on the top of the motor, the tip of the crank, and the pivot of the slider. Since the robot has neither microprocessor nor sensor, we derived the limb configuration θ(t) kinematically by measuring the 3D positions of several optical markers equipped with the links by a motion capture system. The experimental videos are on https://www.youtube.com/watch?v=VzXPOAgaCQU&feature=youtu.be.

In the experiments, we investigate the basic gait pattern and the convergence property of the gait. In order to investigate the convergence property of the limb configuration according to the input voltage, we conducted 84 trials in total, each of which consisted of four trials from three different initial conditions under seven different input voltages ranging from 1.5 to 4.5 V. We set the initial states as follows:

$$\left(a\right)\left(\theta_{\text{LF}},\theta_{\text{RF}},\theta_{\text{LH}},\theta_{\text{RH}}\right)=\left(\pi /2,\pi /2,\pi /2,\pi /2\right),$$

$$\left(b\right)\left(\theta_{\text{LF}},\theta_{\text{RF}},\theta_{\text{LH}},\theta_{\text{RH}}\right)=\left(3\pi /2,\pi /2,3\pi /2,\pi /2\right),$$

$$\left(c\right)\left(\theta_{\text{LF}},\theta_{\text{RF}},\theta_{\text{LH}},\theta_{\text{RH}}\right)=\left(\pi /2,\pi /2,3\pi /2,3\pi /2\right).$$

**FIGURE 6:**
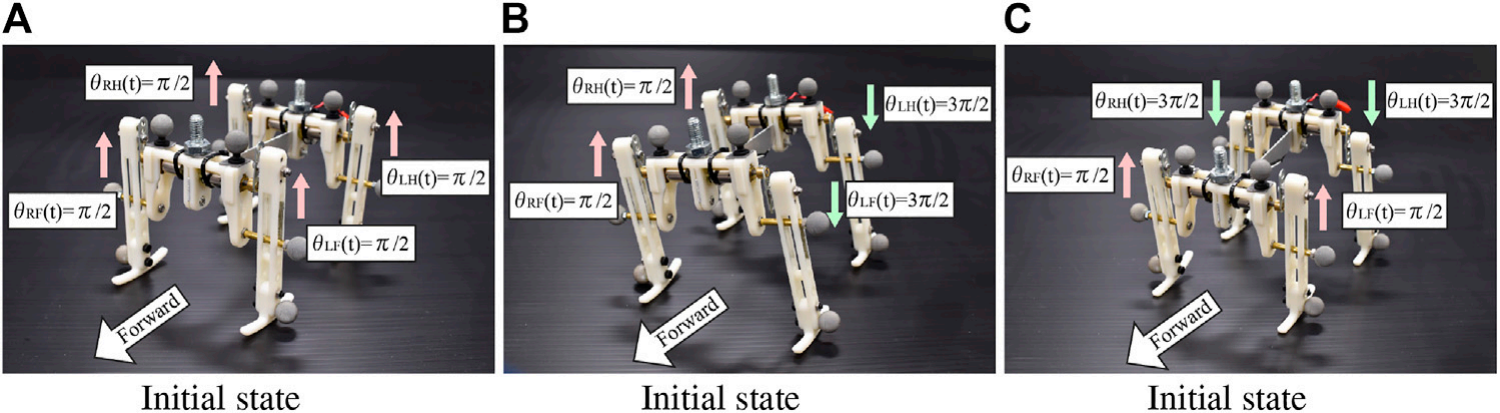
Initial states of the robot. The motor phases are illustrated in the figures.

Figure 6 shows the three initial conditions of motor phases.

### 4.1 Emerged Gaits

**FIGURE 7:**
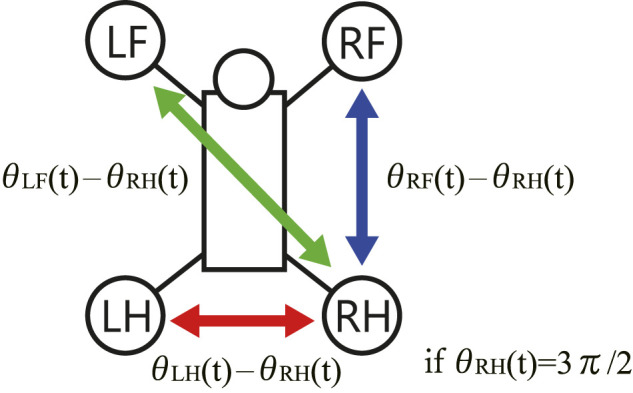
The phase differences between the limbs on the Poincaré section.

In order to investigate the convergence property of limb configuration, the authors visualized the sequence of phase differences of the limbs $\left(\theta_{\text{RF}}-\theta_{\text{RH}},\theta_{\text{LF}}-\theta_{\text{RH}},\theta_{\text{LH}}-\theta_{\text{RH}}\right)$ on Poincaré section when the RH limb are fully extended, namely, when the RH phase is $\theta_{\text{RH}}=\left(2n+3/2\right)\pi ,\left(n=0,1,2,\ldots \right)$ as shown in Figure 7. We show the values ranging from −3π/2 to π/2 in a cyclic manner.

**FIGURE 8:**
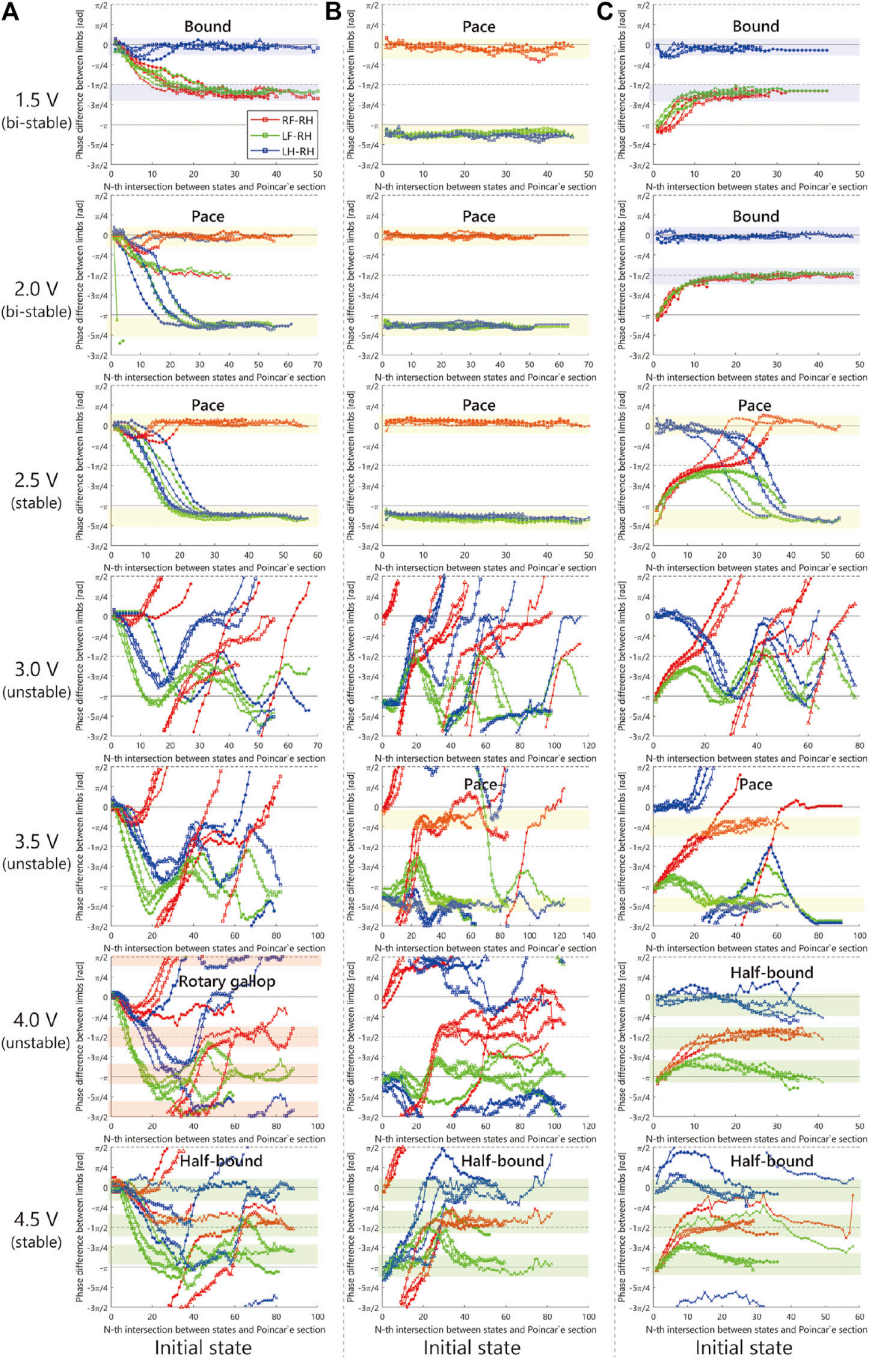
Experimental result.

Figure 8 illustrates the sequences of phase differences of the limbs. The red points denote $\theta_{\text{RF}}-\theta_{\text{RH}}$, the green points are $\theta_{\text{LF}}-\theta_{\text{RH}}$, and the blue points are $\theta_{\text{LH}}-\theta_{\text{RH}}$. The authors qualitatively decided the stability of the limb configuration at each voltage as to whether or not it converged to a constant value with high repeatability. The authors qualitatively determined the gait name in the figure by comparing the typical gait pattern with the limb configuration in the figures and videos.

As shown in Figure 8, when we applied 2.5 V, the limb configuration converged to a pace gait in which the phase difference of front and hind limbs be zero, and with 4.5 V the half-bound gait emerged in which the phase difference of LH and RH limbs be zero. With low voltages such as 1.5 Vand 2.0 V, we observed a bi-stable structure in which the convergence point changes depending on the initial value. Furthermore, note that when 1.5 V is applied, the robot generated a bound gait, in which the phase difference of left and right limbs was small and slightly different from the 4.5 V half-bound. In addition, although most of the gait was unstable from 3.0 to 4.0 V and a rotary gallop gait was observed in the initial state 1) at 4.0 V.

**FIGURE 9:**
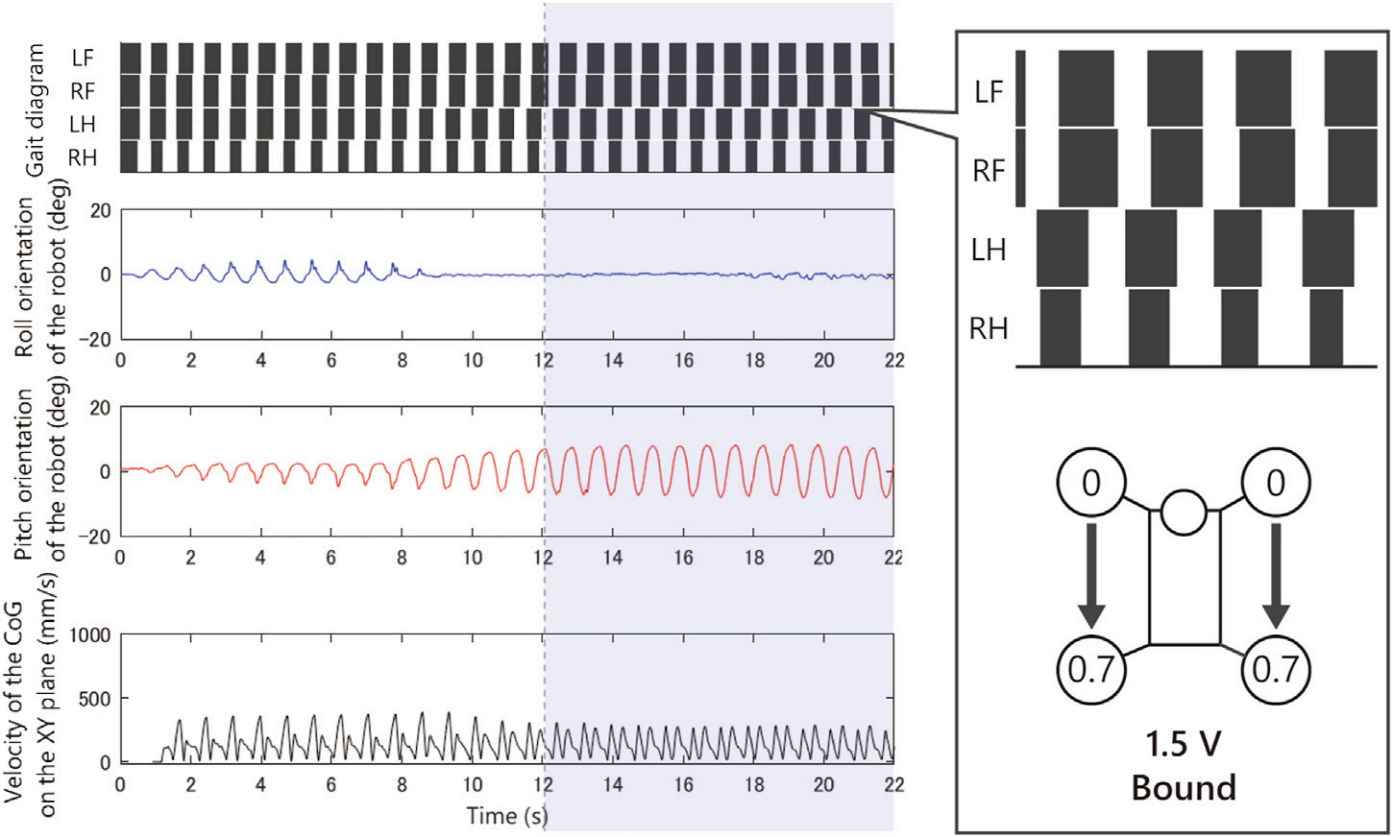
Experimental result with an input voltage of 1.5 V. From the top: gait of the quadruped robot, roll, and pitch orientation. This bound gait emerged from only a few initial values.

**FIGURE 10:**
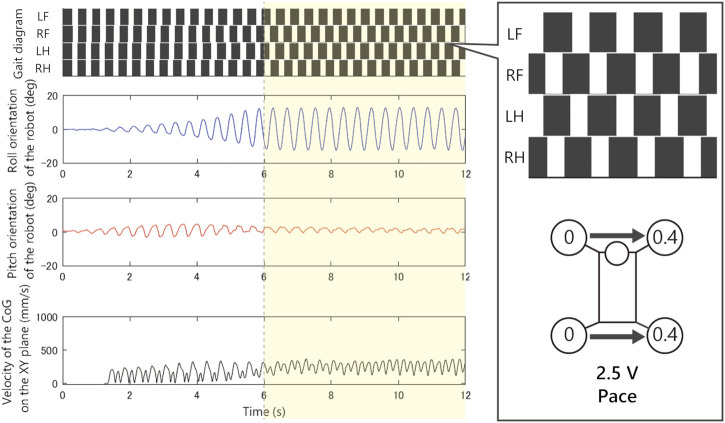
Experimental result with an input voltage of 2.5 V. From the top: gait of the quadruped robot, roll, and pitch orientation. All the trials from the initial values converged to this pace gait.

**FIGURE 11:**
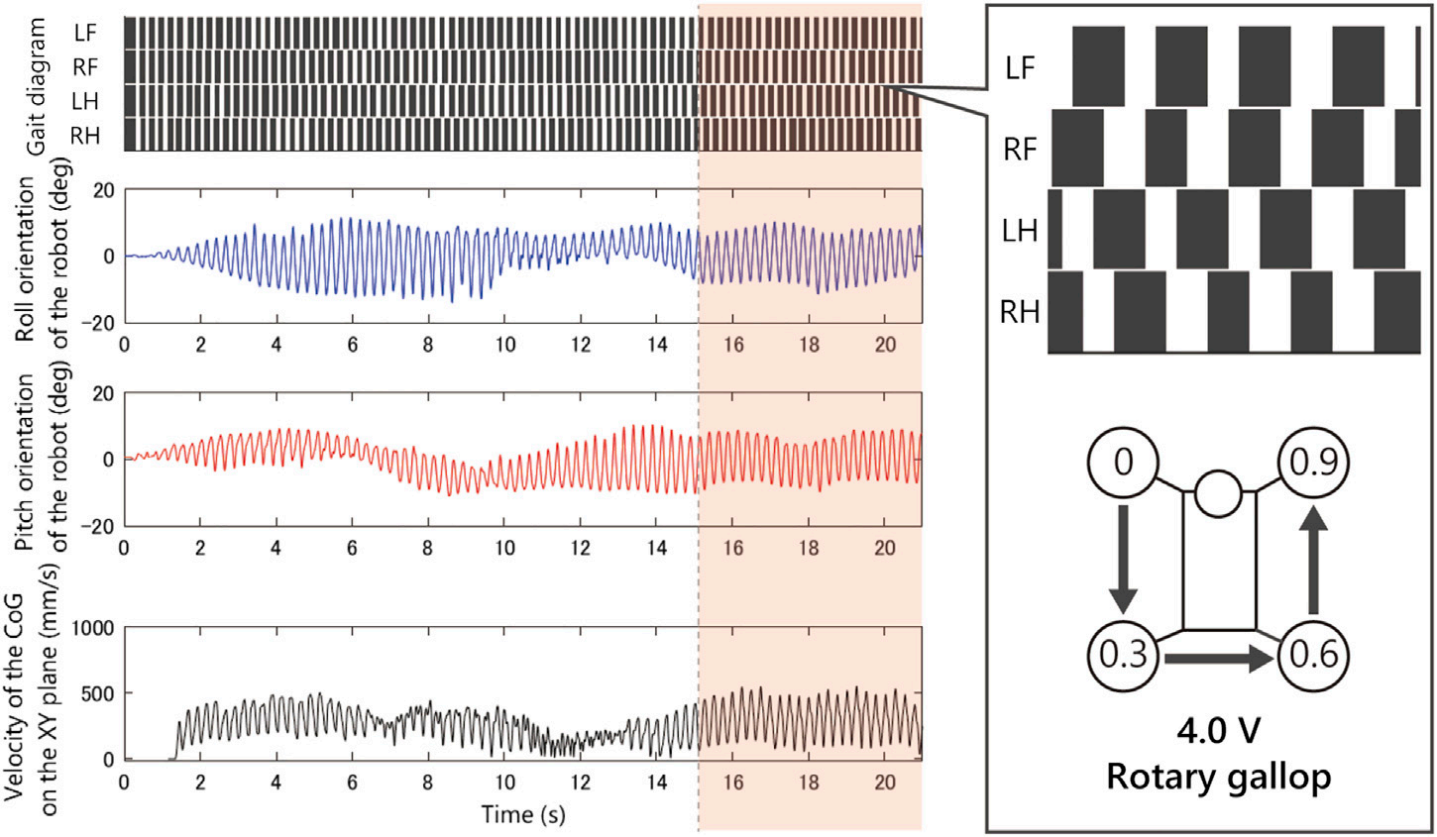
Experimental result with an input voltage of 4.0 V. From the top: gait of the quadruped robot, roll, and pitch orientation. This rotary gallop gait emerged from only a few initial values.

**FIGURE 12:**
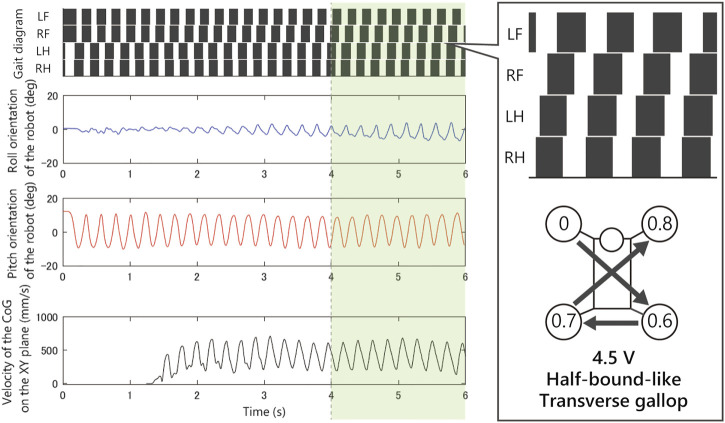
Experimental result with an input voltage of 4.5 V. From the top: gait of the quadruped robot, roll, and pitch orientation. All the trials from the initial values converged to this half-bound-like transverse gallop gait.

Figures 9–12 shows the gait diagram of the quadruped robot, roll, and pitch orientation. The robot has the circular foot to reduce perturbation as much as possible and ground smoothly. Since the ground contact occurs at any point on the circular foot, it is difficult to detect the stance phase by the motion capture system. Therefore, in Figures 9–12, we illustrate the gait chart with a white region, when the leg is contracted 0<sinθ, and a black region, when the leg is extended sinθ≤0. The numbers in the circle indicate the timing when each leg phase becomes θ=π/2, assuming that the moment when $\theta_{\text{LF}}=\pi /2$ is 0 and the next $\theta_{\text{LF}}=\pi /2$ is 1.

As shown in Figure 9, when we applied 1.5 V to the robot, as the bound gait emerges, the side-to-side vibration in roll orientation decreases and the pitch vibration increases. On the contrary, in Figure 10, when we applied 2.5 V to the robot, as the pace gait emerges, the roll vibration increases and the pitch vibration decreases. On the rotary gallop gait in Figure 11, the vibrations increased in both roll and pitch, and on the half-bound-like transverse gallop in Figure 11 keeps the vibration in the roll orientation to a low level.

## 5 Discussion

### 5.1 Comparison With Previous Studies

The experimental results show that the brainless robot generated roughly four types of animal gaits depending on the running speed. These gaits were stabilized, exploiting only the physical interaction between the motor characteristics through the body–environment dynamics. Although some gait generation phenomena using nonneural interaction between the limbs were already reported (McGeer, 1990; Reis et al., 2013; Owaki and Ishiguro, 2017), there is no example of an active walking with multiple motors that can adaptively generate animal-like gait patterns without any sensors or controllers, to the best of the authors’ knowledge. Our results provide a new example of how the actuator and body dynamics alone can generate a variety of gait.

The idea of utilizing the torque–velocity characteristics of a motor for robot control is not completely novel in itself. For example, the concept of back-drivability (Ishida and Takanishi, 2006), which allows a robot’s motion to adapt to the environment, is already reported. The novelty of the phenomenon discovered in this study is that multiple motors interact and synchronize with each other through the physical body and the environment. In other words, the motor, which has been recognized as a mere actuator, actually has the function of a phase oscillator that adjusts the motion pattern of the whole robot body according to the situation. The authors call this function as a phase oscillator, in such a motor the *compliant oscillator* (Masuda et al., 2017a).

Furthermore, the synchronization phenomenon between DC motors may be observed in other types of actuators, such as animal muscles and musculoskeletal robots. The animal muscles have force–velocity characteristics (Kandel et al., 2000), and the pneumatic artificial muscles have force–length characteristics (Klute et al., 1999). In fact, a musculoskeletal robot with pneumatic muscles (Masuda et al., 2020) generated alternating gait patterns of left and right limbs without any computer.

In addition, the motor model Eq. 6 has some interesting similarities with a CPG model proposed by (Owaki and Ishiguro, 2017). Their control law is described as follows:

$$\dot{\theta }\left(t\right)=\omega -\varepsilon N\left(t\right)\text{cos}\theta \left(t\right).$$ (7)

Comparing the motor model Eq. 6 and the controller Eq. 7, the sign and the form of the function of the second term are different. Although the form of the functions are different, they share the same qualitative property of changing the phase speed in response to an external force N(t). Moreover, the two formulas are symmetric because the motor Eq. 6 has the compliant property to external forces, and the controller Eq. 7 has the property of pushing back against external forces. The similarities between the emerged gait patterns of these two equations are interesting and we require further comparison and analysis.

### 5.2 Mechanism of Gait Generation

There are two important factors in understanding this phenomenon. The first factor is the torque–velocity characteristics of the actuator that functions as a feedback controller. The authors think that the dynamics of the motors through the linkage mechanisms Eq. 6 makes the robot generate an adaptive gait. This is because the structure of the robot is extremely simple, and there is no adaptive element other than the motor characteristics. When a limb is supporting the weight of the robot, the phase of the limb stays in place, and when the ground reaction force is decreased as the load is transferred to other limbs, the motor is driven to kick the ground. In other words, when a large external force is applied to the motor from the environment, the motor does not generate inefficient motion against the large external force. And after the peak of the external force has passed, the motor sends momentum to the body with a slight phase delay, and large-amplitude motion is effectively generated.

The second important factor of the phenomenon is the vibration mode intrinsic in the robot body. In the author’s previous work (Masuda et al., 2017a), we have analyzed the synchronization mechanism of motors in more fundamental systems, such as spring–mass systems. The experiments and simulations in the article show that the synchronized DC motors converge to the resonant mode of the system and that the motors generate the resonant modes (primary, secondary, and tertiary modes) by increasing the input voltage. Although the system in this study has many nonlinearities, the basic effects that the motor brings to the system are not very different from those of a linear system.

In the experiment Figures 8–12 with 1.5 V, at least two stable periodic solutions exist. As the voltage increased from 2.0 to 2.5 V, the bound gait was not observed, and the convergence property to the pace gait improved. And as the voltage was increased further, the pace gait disappeared from 4.0 to 4.5 V, and the gallop and half-bound gaits with a pitch oscillation emerged. From the results and analogy with phenomena observed in the previous study by Masuda et al. (2017a), we hypothesized that the phenomenon of the gait selection from pace to gallop could be interpreted as the frequency of the robot motion, which left the resonant frequency of the side-to-side motion and approached the resonant frequency of the gallop modes due to the increase in voltage. In other words, the robot body has a few vibration modes similar to the gait of an animal, and that these modes are entrained by the rotating motor with the force–velocity characteristics. We expect that a similar phenomenon may occur in the body of an animal.

### 5.3 Discussion of the Individual Gaits Emerged

Animals generally walk at slow speed and bound for high speed. However, the robot showed bound at slow speed. Although it is unclear why bound occurs when a low voltage is applied, we think that it is difficult to propel the body with the torque of one leg when the applied voltage is extremely low, so the leg stops until the phases of both legs become equal.

Moreover, the robot did not generate trot gait. The mechanism by which trot gait did not occur is also unclear. However, the previous study using a CPG with similar dynamics to our model (Owaki and Ishiguro, 2017) generated walk, trot, and gallop. Therefore, the authors think the first step to understanding the mechanism is to compare in detail the effects of this control law and the motor dynamics used in this study.

Notably, the asymmetric gaits appeared from the left–right symmetric robot. We expected that if the physical properties of the robot were perfectly symmetric, then either symmetric gaits would arise, or it would diverge into two types of gait (left-lead and right-lead). However, the robot generated asymmetric gaits (rotary gallop and half-bound-like transverse gallop) in the experiments. Although the mechanism that causes the convergence to asymmetric solutions is still unclear, we expect that the system is sensitive to small asymmetric errors such as individual differences of the motors, and these asymmetric errors cause the solution to converge to the asymmetric gaits.

### 5.4 Expected Application

We also expect synchronization between the motors to be applied as a novel control method for real-world robots. Modeling and controlling complex nonlinear systems, such as soft robots and legged robots, is very difficult. In the motion generation approach introduced in this study, some actuators embedded in the robots’ whole body react immediately to stimuli from the outside world and produce natural movement by harmonizing the body–environment dynamics. This idea would be a new approach to robot design, embedding a software-free controller throughout the body to generate adaptive whole-body movements without control.

## 6 Conclusion

In this study, we reported an example of how the actuator and body dynamics alone can generate a variety of animal gait. Although this robot does not have any sensors or microprocessors, the motors adjust their phases autonomously and finally converged to a steady gait pattern. Furthermore, by raising the input voltage from the power supply, various gaits (pace, bound, rotary gallop, and half-bound-like transverse gallop) were observed. We investigated the convergence property of the gaits for several initial states and input voltages, and described detailed experimental results of each gait observed. The analogy between the results and the previous analysis in the work by Masuda et al., 2017a suggested that the emerged gaits may be a kind of resonant mode intrinsic in the robot body.

## Data Availability

The original contributions presented in the study are included in the article/Supplementary Material; further inquiries can be directed to the corresponding author.

## Footnotes

1 Note that, although $J,D,K_{T}$, and $K_{E}$ are also constants that depend on the gear ratio *n*, we omitted them for simplicity.

2 In most motors, the inductance and viscous resistance coefficient are kept small. For the inertia term, the rotor and shaft with gears were only 2 g and 1 g.

3 Although the dynamics of the limb linkage also exists during the stance phase, we ignore its influence because the weight of the limb linkage is small compared to the body weight, and the displacement of the center of gravity of the limb linkage is very small because the toes are fixed to the ground.

4 From −π/2≤θ(t)<π/2 and Figure 4, we get sin(ϕ)>0. Moreover, we get cos(ϕ)>0 from a constraint of the slider crank mechanism −π/2<ϕ(t)<π/2. Therefore, sin(2ϕ)=2sin(ϕ)cos(ϕ)>0.

5 At the equilibrium point $\dot{\theta }\left(t\right)=0$, the limb angle satisfies ϕ(t)=1/2asin2ω/εbN(t). Therefore, when N(t)≫0, the limb angle becomes ϕ(t)≈0. Moreover, when ϕ(t)≈0, from the geometric constraint in Figure 4, the motor angle becomes θ(t)=π/2 or 3π/2. Finally, from the discussion so far, we found a stable equilibrium point θ=π/2 and an unstable equilibrium point θ=3π/2.
